# Radiofrequency Microneedling With 1927 nm Thulium Laser Versus Radiofrequency Microneedling Monotherapy for Rejuvenation of Photoaged Skin

**DOI:** 10.1111/jocd.70685

**Published:** 2026-01-21

**Authors:** Lynhda Nguyen, Marco Blessmann, Stefan W. Schneider, Katharina Herberger

**Affiliations:** ^1^ Department of Dermatology and Venereology University Medical Center Hamburg‐Eppendorf Hamburg Germany; ^2^ Department of Plastic, Reconstructive, and Aesthetic Surgery University Medical Center Hamburg‐Eppendorf Hamburg Germany

**Keywords:** pigmentation, radiofrequency microneedling, skin rejuvenation, skin tightening, thulium laser

## Abstract

**Background:**

The combination of radiofrequency microneedling (RFMN) with fractional 1927 nm thulium (Tm) laser has gained popularity in clinical practice. However, comparative studies remain limited.

**Objectives:**

To evaluate the efficacy and safety of combined RFMN/Tm laser treatment versus RFMN alone for rejuvenation of photoaged skin of the lower face and neck area.

**Material and Methods:**

A prospective, evaluator‐blinded, clinical study. Patients were assigned to a combined protocol consisting of 1–3 RFMN treatment sessions immediately followed by Tm laser, supplemented by three additional Tm laser sessions at 6–8‐week intervals. Study endpoints were compared with data from our previously published RFMN monotherapy cohort. Outcomes were assessed using validated clinical grading scales, computer‐assisted analysis of submental volume, and a standardized color space system to quantify skin tone evening. Pain was assessed using a numeric rating scale. Adverse events (AEs) were monitored throughout the study period.

**Results:**

26 received the combined treatment, and 27 patients were treated with RFMN alone. Mean ages were 50.7 ± 7.4 and 55.6 ± 8.9 years, respectively. Both groups showed significant reductions in submental volume without significant differences between them. However, only the combination group demonstrated a distinct improvement in hyperpigmented lesions, as quantified by the color analysis. Blinded evaluations noted improvements in the melomental area, jawline, and neck in both groups, while greater improvements in skin tone evening and therefore overall appearance were seen only in the combination group. No serious AEs were reported.

**Conclusion:**

Both treatment modalities effectively addressed lower facial and neck laxity. The addition of Tm laser to RFMN provided additional benefits in treating pigmentary irregularities and improving overall clinical outcome without notably compromising downtime. Larger studies are warranted to validate these findings and assess long‐term outcomes.

**Trial Registration:**

This study was preregistered in clinicaltrials.gov (NCT06029725) on 23 August 2023

## 1 Introduction

Radiofrequency microneedling (RFMN) is a well‐established technique in aesthetic dermatology, recognized for its efficacy in improving skin laxity, fine lines, and overall dermal texture [1, 2]. The procedure delivers radiofrequency energy via insulated microneedles into the dermis in a controlled manner, thereby stimulating neocollagenesis and promoting dermal remodeling while minimizing disruption to the epidermis [2, 3, 4].

In recent years, there has been growing interest in combining RFMN with fractional laser technologies, particularly the 1927 nm thulium (Tm) laser. The Tm laser, which uses water as its primary chromophore, selectively targets the epidermis. It has demonstrated efficacy in treating superficial pigmentary changes and textural irregularities [5, 6]. The rationale behind this combined approach is to leverage the effects of both modalities, addressing deeper dermal concerns with RFMN and more superficial epidermal changes with the Tm laser within a single treatment protocol.

Although this treatment approach is applied in daily practice, there is limited data on its efficacy and safety compared to RFMN monotherapy. Furthermore, it is unclear whether the Tm laser has a long‐term effect on the skin. Therefore, this study aims to evaluate the efficacy, safety profile, and patient‐reported outcomes of RFMN alone versus an RFMN/Tm laser treatment.

## 2 Material and Methods

### 2.1 Study Design

This study was designed as a prospective, evaluator‐blinded, clinical study. It was approved by the local ethics committee (2023‐101110‐BO‐ff), conducted in accordance with the declaration of Helsinki, and preregistered on ClinicalTrials.gov (NCT06029725). Participants were assigned to a combination treatment consisting of RFMN and 1927 nm Tm laser. Follow‐up visits were conducted 3 and 6 months post‐treatment. Study endpoints were compared with data from a previously published cohort from our group that had undergone RFMN monotherapy [1]. These data were reanalyzed for the present investigation. The primary outcome measure was the change in submental area volume between baseline and 6 months post‐treatment as an indicator of skin tightening. Additionally, clinical evaluations were performed using numeric assessment scales and standardized photographic documentation.

### 2.2 Inclusion and Exclusion Criteria

Female and male patients aged 35 years and older were included in the study if they showed photoaging signs with skin laxity, wrinkles, or fine lines of the lower face and neck area. Patients were excluded if they had undergone surgical procedures within the past 2 months, received injectable treatments within the last 4 weeks, or had a known tendency for excessive scarring. Further exclusion criteria included significant scarring or lesions in the treatment area. Additionally, patients who experienced a change in body weight > 5% compared to their baseline measurement by the final control visit were excluded from the volume assessment.

### 2.3 Treatment Protocol

Before each treatment session, patients received a topical anesthetic ointment (containing 23% lidocaine, 3.5% tetracaine, and 3.5% tetracaine‐HCl) applied under occlusion for at least 60 min. The anesthetic was then thoroughly removed using a disinfectant solution (Octenisept, Schülke & Mayr GmbH, Norderstedt, Germany) prior to treating the designated areas. Depending on the group assignment, patients underwent 1–3 treatment sessions with insulated radiofrequency microneedling (RFMN; Genius, Cynosure Lutronic, Seoul, South Korea), administered either as monotherapy or followed immediately by a Tm laser session (LaseMD Ultra, Cynosure Lutronic, Seoul, South Korea). Participants assigned to the RFMN/Tm laser group additionally received three standalone Tm laser treatments. Treatment sessions were scheduled at 6–8 week intervals. Immediately after Tm laser application, a vitamin A serum (VA serum, Cynosure Lutronic, Seoul, South Korea) specifically developed for use with this device was applied in accordance with the manufacturer's protocol. Patients were instructed to reapply the serum every 2 h until the ampoule was exhausted, typically within 2–3 days post‐treatment. Treatment parameters are summarized in Table 1. In cases of high pain intensity, fluence was adjusted. Continuous air cooling was provided during the procedure (Cryo 6, Zimmer MedizinSysteme GmbH, Neu‐Ulm, Germany).

**TABLE 1:** Treatment parameters for radiofrequency microneedling (RFMN) for both the RFMN monotherapy group and the combined RFMN/Thulium (Tm) laser group, focusing on the lower face, jawline/submental region, and neck.

Device	Passes	Handpiece/tip	Lower face	Jawline/submental	Neck
RFMN	1st	7 × 7 pins, 1.4 mm pitch between needles, 50%–70% overlap	1.5 mm 34 mJ/pin	2.3 mm 50 mJ/pin	1.8 mm 34 mJ/pin
	2nd		1.2 mm 26 mJ/pin	1.9 mm 50 mJ/pin	1.5 mm 30 mJ/pin
	3rd		1.0 mm 18 mJ/pin	1.5 mm 40 mJ/pin	1.0 mm 26 mJ/pin
Thulium laser	6 passes in total	~100 μm spot size, 5% coverage	40 beams/cm^2^, 20 W, 20 J/cm^2^ per pass		

*Note:* In participants allocated to the combination group, the Tm laser was administered according to the parameters outlined above.

### 2.4 Standardized Photo Documentation

Photo documentation was performed using a three‐dimensional imaging system (Vectra^H2^, Canfield, Bielefeld, Germany) with a neutral background and a single ring light. To ensure a standardized positioning, the bipupillary and Frankfurt planes were aligned using a laser spirit level (PLL 1 P, Bosch, Stuttgart, Germany). Patients were instructed to maintain a relaxed facial expression with closed mouths. Images were captured from the front and both 45° side angles.

### 2.5 Volume Measurement

Submental volume changes between baseline and the follow‐up visit at 180 days post‐treatment were assessed using the Vectra Analysis Module software (VAM, Canfield, Bielefeld, Germany). Anatomical landmarks including the mentum, mandibular angles, sternocleidomastoid muscles, and laryngeal prominence were defined as reference points for the submental region [7]. Volume differences were calculated using the software's integrated analysis algorithm.

### 2.6 Colorimetric Assessment

For objectifying the evening of skin tone, the CIEL*a*b* (Commission Internationale de l'Erclairage) color space system was employed, a numerical three‐dimensional color space adapted to human perception [8]. The L* value represents the luminosity of colors and correlates with the severity of dyspigmentation. Dyspigmentation was quantified by calculating the ΔL* in both before and after images, which is the difference between the L* value of unaffected skin and the L* value of hyperpigmented skin. Computer‐assisted tracing was used to ensure measuring identical spots.

### 2.7 Clinical Assessment

Clinical assessment was performed by an investigator and an independent, blinded, board‐certified plastic surgeon. The melomental region, jawline, neck, pigmentation/lentigines, and overall improvement were evaluated using a scale from 0 (very much improved) to 4 (worse), based on the Global Aesthetic Improvement Scale (GAIS) and photo documentation. In addition, patients were asked about their overall improvement using the GAIS as well as about their treatment satisfaction using a scale from 0 (not at all) to 4 (very).

### 2.8 Safety and Tolerability

During each treatment session, patients were asked to rate their pain intensity on a numeric scale from 0 (no pain) to 10 (unbearable pain). In the event of any adverse events (AEs), both written and photographic documentation were obtained, and appropriate medical management was provided as needed.

### 2.9 Statistical Analysis

Statistical analysis was conducted using Microsoft Excel (version 16.95.4, Microsoft Corporation, Redmond, WA, USA) and SPSS (version 29.0.2.0, IBM Corporation, Armonk, NY, USA). Unless otherwise specified, group differences in means were assessed using the unpaired *t*‐test. To compare the ΔL* before and after treatment, the Mann–Whitney‐Wilcoxon test was applied. A *p*‐value of < 0.05 was considered statistically significant. Descriptive data are presented as means, standard deviations, and ranges (minimum—maximum).

## 3 Results

### 3.1 Baseline Characteristics

In the RFMN group, 30 female patients were initially enrolled, with a mean age of 55.9 ± 8.7 years. One patient was excluded due to undergoing jawline augmentation during the study period. Two additional participants had body weight changes of > 5% and were therefore excluded from the volumetric analysis.

In the combination treatment group, 26 female participants were included and completed the study. The mean age was 50.7 ± 7.4 years. None of the participants reported body weight changes of > 5% compared to baseline. Details on baseline data are shown in Table 2.

**TABLE 2:** Patient characteristics.

	RFMN group	RFMN/Tm laser group
Sex (female/male)	27/0	26/0
Age (mean ± SD) (min.–max.)	55.6 ± 8.9 years (38–75)	50.7 ± 7.4 years (35–69)
Fitzpatrick skin type	I (2); II (18); III (6); IV (1)	I (6) II (10); III (8); IV (2)
Number of RFMN sessions Mean ± SD (min.–max.)	1 session: 13 2 sessions: 12 3 sessions: 1 1.59 ± 0.64 (1–3)	1 session: 12 2 sessions: 11 3 sessions: 3 1.65 ± 0.7 (1–3)
Energy per session (mean ± SD) (min.–max.)	892.3 ± 409.13 (248.3–2956.4)	RFMN: 1315.51 ± 345.92 (365.2–1901.4) Thulium: 878.65 ± 413.92 (267–2100)
Total energy (mean ± SD) (min.–max.)	1518.2 ± 784.1 J (532.4–3075.5)	RFMN: 1953.68 ± 910.28 (595.9–3599.4) Thulium: 2005.14 ± 788.54 (880–3580)

### 3.2 Volume Changes

**FIGURE 1:**
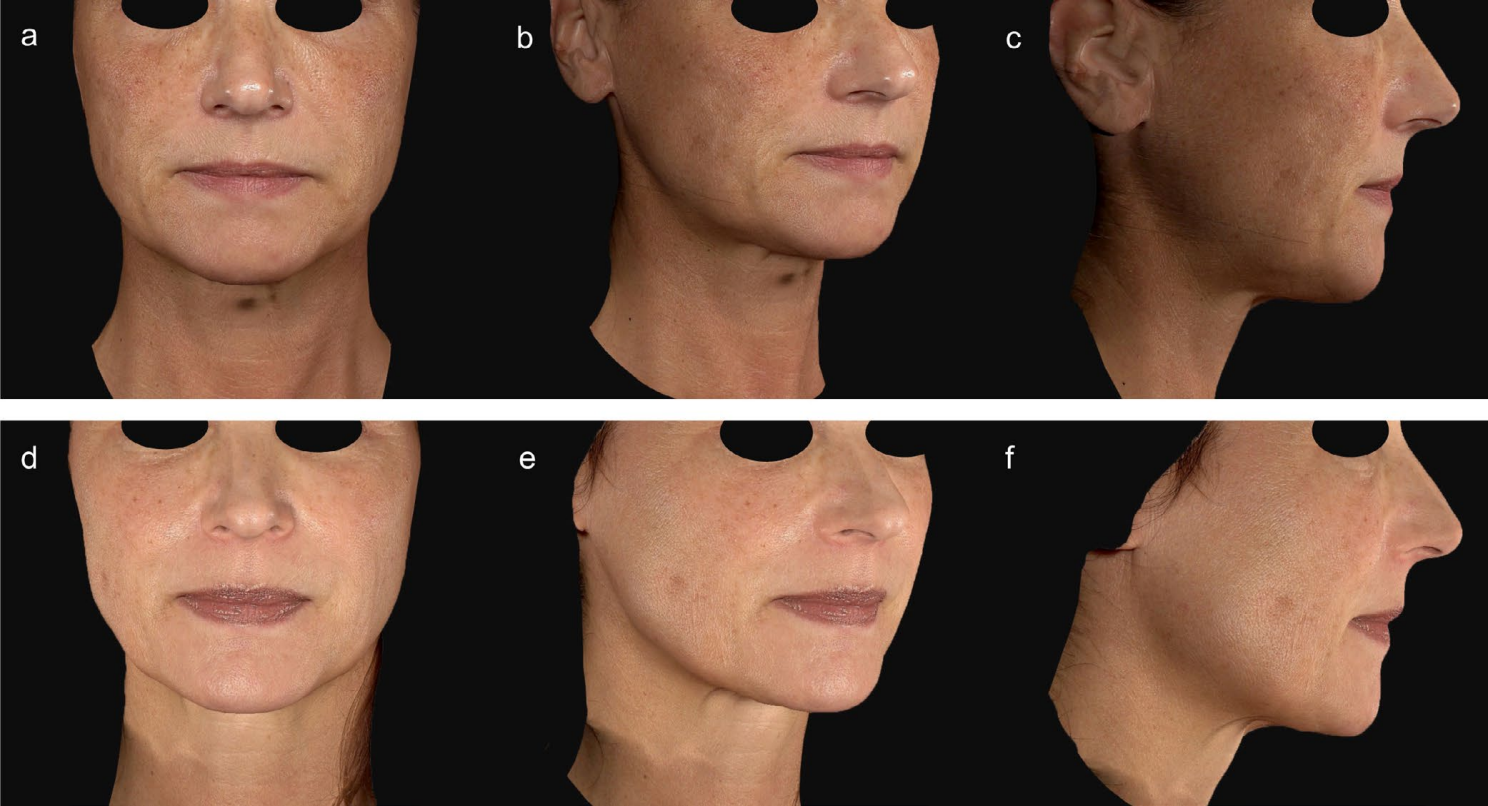
Photographic documentation at baseline (a–c) and 180 days post‐treatment (d–f) following a combined treatment of two radiofrequency microneedling/thulium laser sessions and three consecutive thulium laser treatments of the lower face and neck area. The patient demonstrated visible skin tightening in the submental area along with a marked reduction in pigmentation irregularities.

**FIGURE 2:**
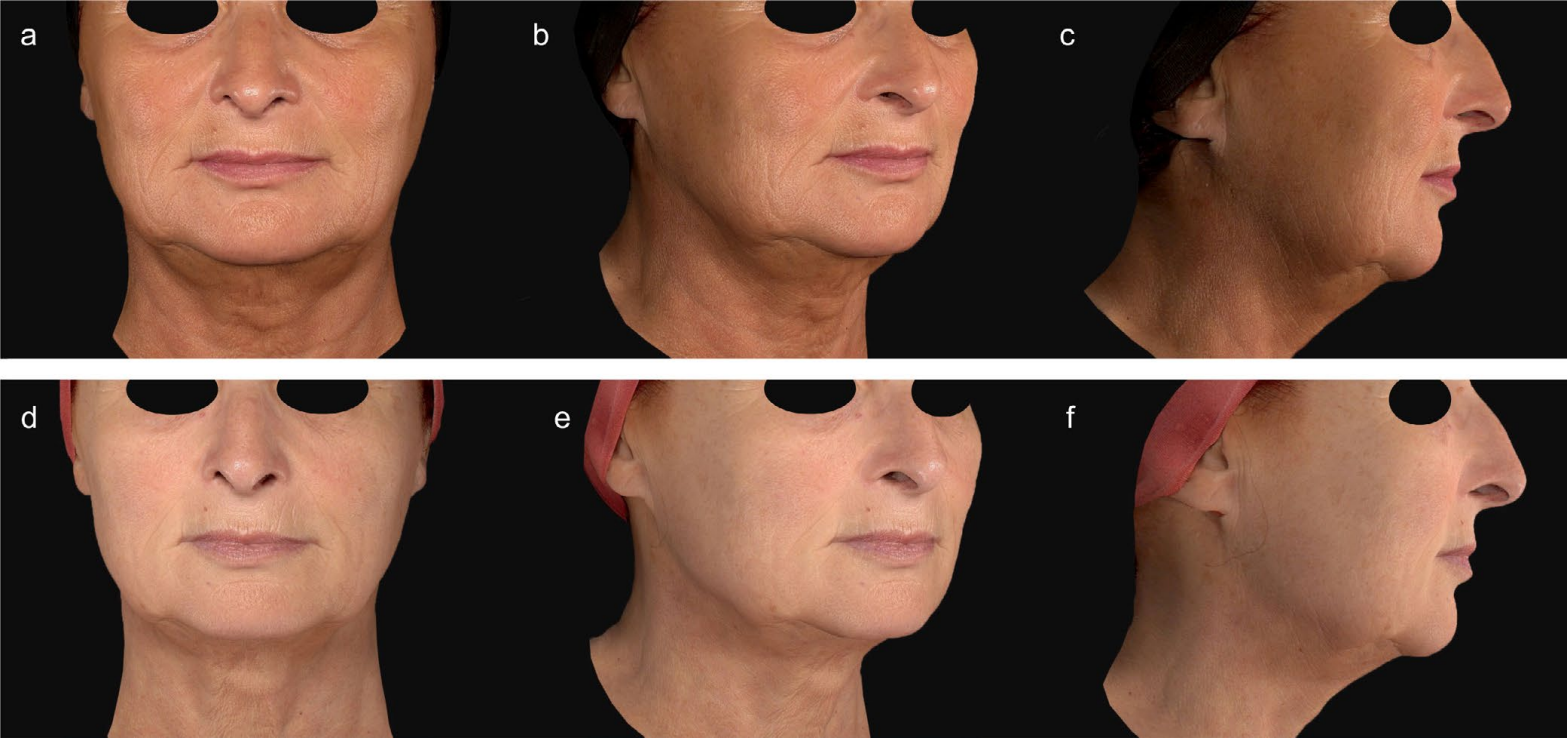
Clinical images before (a–c) and 180 days after (d–f) three radiofrequency microneedling treatment sessions of the lower face and neck area. The patient is shown from the front, at 45°, and in profile from the right side. A notable reduction in submental volume was observed. However, there was no significant improvement in hyperpigmented lesions. It should be noted that the baseline images were taken during summer, which may account for the patient‘s tanned appearance.

At 180 days post‐treatment, both groups demonstrated a significant reduction in submental volume: −4.72 ± 10.07 cm^3^ (−26.65 to 16.01; *p* < 0.05) in the RFMN group and −6.06 ± 7.73 cm^3^ (−19.25 to 14.85; *p* < 0.05) in the RFMN/Tm laser group. There was no statistically significant difference between the two groups (*p* = 0.606). Figures 1 and 2 illustrate the clinical outcomes before treatment and 180 days following both treatment approaches.

### 3.3 Colorimetric Analysis

**FIGURE 3:**
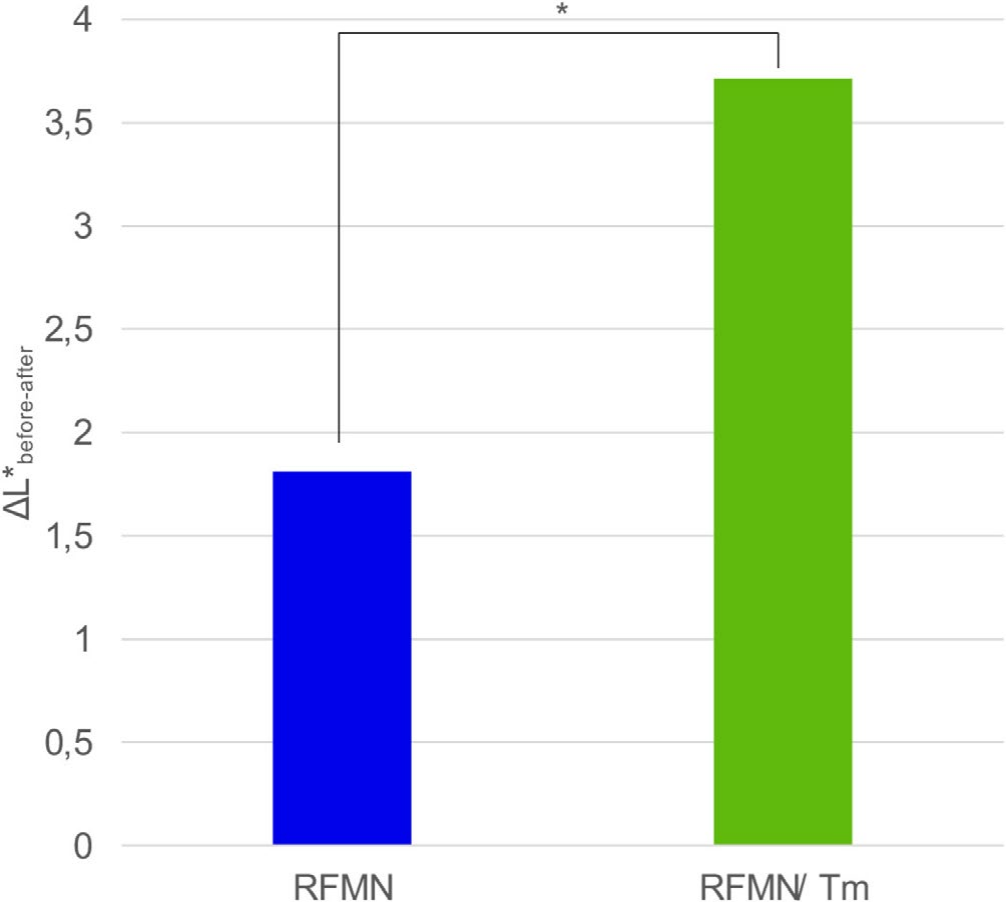
ΔL* value represents the skin tone difference between hyperpigmented lesions and unaffected skin at baseline and 180 days after the final treatment session. The combination of radiofrequency microneedling and thulium laser resulted in a significantly greater improvement in skin tone compared to radiofrequency microneedling alone.

To assess changes in dyspigmentation, we conducted an objective evaluation using a standardized color space system. The ΔL* value, representing the luminosity difference between unaffected and hyperpigmented skin, decreased from 8.12 ± 3.54 (2.19–18.07) to 4.49 ± 2.14 (1.01–9.35) following RFMN/Tm laser treatment, indicating a notable improvement in skin tone (*p* < 0.05). In the RFMN group, ΔL* values changed from 6.35 ± 3.86 (1.56–19.99) to 4.91 ± 2.79 (0.86–9.12) (*p* > 0.05). Figure 3 shows that the clearance rate of the complimentary Tm laser treatment is significantly higher than with RFMN treatment only.

### 3.4 Clinical Assessment

**FIGURE 4:**
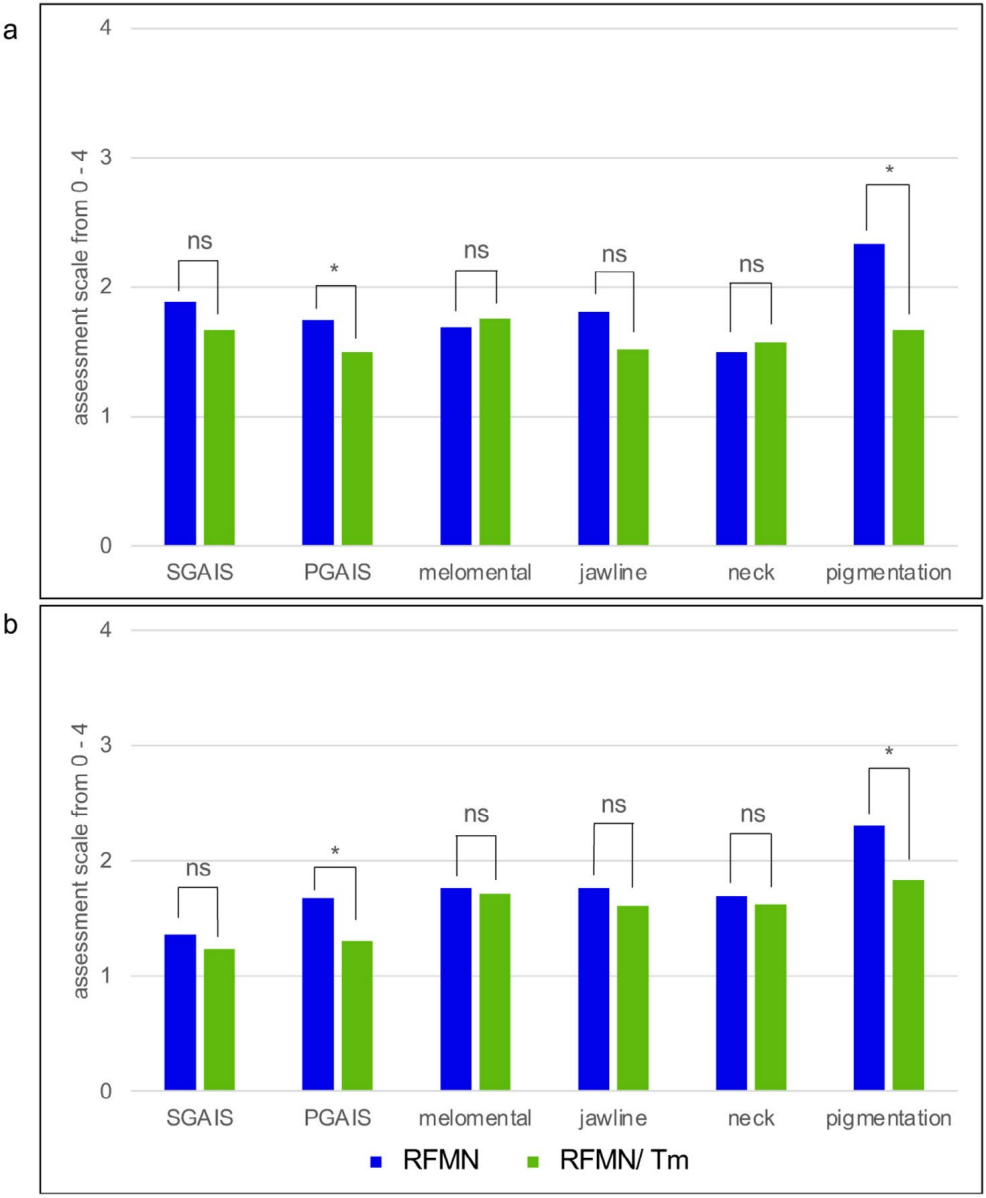
Clinical outcomes following radiofrequency microneedling (RFMN) and combined RFMN/thulium (Tm) laser treatment at day 90 (a) and day 180 (b) post‐treatment, as assessed by patients (Subject Global Aesthetic Improvement Scale, SGAIS) and physicians (Physician Global Aesthetic Improvement Scale, PGAIS). In addition, improvement of the melomental region, jawline, neck, and hyperpigmentation was assessed on a 5‐point scale (0 = very much improved; 4 = worsened). *p < 0.05; ns = not significant.

Clinical assessments by the treating physician and an independent, blinded investigator demonstrated noticeable improvements in overall appearance, as well as the melomental region, jawline, neck, and dyspigmentation. In both groups, outcomes were more favorable at 180 days post‐treatment compared to 90 days post‐treatment. Furthermore, the addition of Tm laser therapy led to a significant improvement in hyperpigmented lesions and overall aesthetic results compared to treatment without Tm laser. These findings are further detailed in Figure 4.

### 3.5 Patient Satisfaction

Patient satisfaction scores averaged 3.5 ± 0.7 (2–4) in the RFMN and 3.6 ± 0.6 (2–4) in the RFMN/Tm laser group, with no significant difference between them (*p* > 0.05).

### 3.6 Safety and Tolerability

Pain intensity for RFMN treatment was reported as 5.6 ± 1.9 (2–8) in the RFMN group and 4.8 ± 1.5 (2–8) in the RFMN/Tm laser group (*p* < 0.05). For the Tm laser alone, pain was rated at 3.8 ± 2.0 (0–8). In the RFMN group, four patients developed perioral dermatitis, which resolved spontaneously within a few days. One of these patients also experienced a hematoma near the orbital rim. In the RFMN/Tm laser group, one patient reported post‐inflammatory hyperpigmentation at the lower face which resolved after the second Tm laser treatment. Another patient reported swelling that lasted for 7 days. As a result, the energy settings for subsequent Tm laser treatments were reduced. All patients experienced transient erythema and swelling immediately after treatment, which resolved within a few days. No serious AEs were reported throughout the study (Table 3).

**TABLE 3:** Patient satisfaction (ranging from 0 (not at all) to 4 (very)), pain intensity (ranging from 0 (no pain) to 10 (unbearable pain)), and safety in the RFMN and RFMN/Tm laser group.

	RFMN group	RFMN/Tm laser group
Patient satisfaction (mean ± SD) (min.–max.)	3.5 ± 0.7 (2–4)	3.6 ± 0.6 (2–4)
Pain intensity (mean ± SD) (min.–max.)	5.6 ± 1.9 (2–8)	4.8 ± 1.5 (2–8)
Adverse events	4 patients: perioral dermatitis 1 patient: hematoma	1 patient: post‐inflammatory hyperpigmentation 1 patient: swelling for 7 days

## 4 Discussion

The present study evaluated the efficacy and safety of RFMN alone compared to a combined treatment of RFMN and Tm laser using a clinical, comparative, and evaluator‐blinded study design. Both subjective clinical assessments and objective, computer‐assisted analyses were employed to comprehensively assess the comparative outcomes of the two therapeutic approaches.

Both treatment groups demonstrated a statistically significant improvement in skin tightening, reflected by a measurable reduction in submental volume. However, three‐dimensional volumetric analysis did not show a statistically significant difference between groups, despite a slightly greater volume reduction in the combination group. This difference appears to be attributable to the higher final RFMN energy applied. After recognizing that increased energy levels may lead to greater skin tightening, the RFMN settings were adjusted accordingly in the combination group, which was treated later in the study [9]. These results suggest that while both RFMN and combined RFMN/Tm laser treatments are effective for skin tightening, the addition of Tm laser does not provide an additional volumetric benefit beyond what is achieved with RFMN alone.

Clinical assessments indicated that the combined treatment offered additional benefits beyond volumetric reduction. Participants in the RFMN/Tm laser group showed significant improvements in the overall aesthetic outcome. These enhancements are likely attributable to the fractional Tm laser, which targets more superficial skin layers [10]. Growing evidence supports the use of non‐ablative fractional lasers to facilitate the delivery of topical formulations [11, 12, 13]. The microthermal zones created by the Tm laser may enhance the transdermal delivery of topically applied agents, such as a vitamin A serum used in this study, potentially contributing to additional clinical effects [14, 15].

Consistent with previous research, our clinical assessments indicated ongoing collagen remodeling even months post‐treatment [16, 17, 18]. However, the extent of improvement varied considerably among participants, with some showing minimal or no noticeable changes. We hypothesize that this variability may be attributed to differences in baseline skin condition, as individuals with more pronounced skin laxity and hyperpigmentation tended to exhibit comparatively greater improvements. Due to the limited sample size, a meaningful subgroup analysis was not feasible. Patient satisfaction was comparable between groups. Further split‐face studies are warranted to clarify these findings.

Tolerability was another focus of this study, particularly in the context of optimizing patient comfort during aesthetic procedures. In the RFMN‐only group, patients reported moderate to high pain levels. To reduce discomfort in the combination therapy group, the application time of topical anesthetic ointment was extended to 60 min, which led to a noticeable reduction in pain during the RFMN procedure. However, this adjustment was introduced later in the study for the RFMN/Tm laser group, limiting direct comparison. Prior to the Tm laser treatment, the anesthetic was fully removed, resulting in discomfort, despite the Tm laser typically being well tolerated when used alone. Consequently, laser fluence had to be reduced to maintain patient comfort.

All patients experienced transient swelling and erythema immediately following treatment. In the RFMN group, four patients developed perioral dermatitis, and one patient presented with a hematoma at the orbital rim. In the RFMN/Tm laser group, one case of post‐inflammatory hyperpigmentation occurred at the lower face after the initial RFMN session, which resolved following the second Tm laser treatment. No serious AEs were observed throughout the study period.

This study has some limitations. First, the sample size was limited, which reduces the ability to generalize the results to broader patient populations. Second, the study cohort was relatively homogeneous, as most patients in our routine clinical practice presented with Fitzpatrick skin types I–III. This may restrict the applicability of the findings to more diverse populations. Finally, internal and external influencing factors—such as lifestyle, skin care habits, and UV exposure—could not be accounted for in the outcome analysis. Future studies with larger, more diverse cohorts and randomized controlled designs are needed to validate and expand upon these findings. In addition, the protocol incorporated three supplementary thulium laser sessions following the initial combination treatment, reflecting manufacturer recommendations and common clinical practice at the time of study design. While this approach enhances clinical relevance, it precludes an unbiased comparison between the immediate effects of the combination therapy alone and those of subsequent thulium laser monotherapy. Future prospective studies with separate monotherapy arms are warranted to better delineate the individual and interactive effects of these modalities.

In conclusion, both treatment modalities demonstrated significant efficacy in improving skin laxity of the lower face, jawline, and neck. The combination of RFMN and Tm laser additionally showed enhanced effects on superficial signs of photoaging, such as skin tone and texture, suggesting an additional benefit. Further research is warranted to confirm these clinical outcomes in larger, diverse patient populations and to explore long‐term efficacy and safety. In particular, histologic assessments could provide deeper insight into the mechanisms underlying tissue remodeling and pigmentary changes observed after treatment. Moreover, split‐face studies could offer a more direct comparison of RFMN versus combined RFMN/Tm laser therapy in terms of clinical efficacy and patient satisfaction.

## Funding

During the study period, the radiofrequency microneedling device (Genius) and the thulium laser (LaseMD Ultra) were provided by Cynosure Lutronic.

## Ethics Statement

The study was approved by Hamburg ethics committee (2023‐101110‐BO‐ff) and conducted in accordance with the declaration of Helsinki. It was preregistered on ClinicalTrials.gov [NCT06029725].

## Consent

The patients in this manuscript have given written informed consent to publication of their case details.

## Conflicts of Interest

The authors declare no conflicts of interest.

## Data Availability

The data that support the findings of this study are available from the corresponding author upon reasonable request.
